# Premature termination, satisfaction with care, and shared decision-making during home treatment compared to inpatient treatment: A quasi-experimental trial

**DOI:** 10.1192/j.eurpsy.2023.2443

**Published:** 2023-09-08

**Authors:** Stefan Weinmann, Konstantinos Nikolaidis, Gerhard Längle, Sebastian von Peter, Peter Brieger, Jürgen Timm, Lasse Fischer, Svenja Raschmann, Martin Holzke, Julian Schwarz, Luisa Klocke, Sandeep Rout, Constanze Hirschmeier, Uwe Herwig, Janina Richter, Reinhold Kilian, Johanna Baumgardt, Johannes Hamann, Andreas Bechdolf

**Affiliations:** 1 Department of Psychiatry, Psychotherapy and Psychosomatic Medicine, Hospital an der Lindenhöhe, Offenburg, Germany; 2 University Psychiatric Hospital Basel, University of Basel, Basel, Switzerland; 3 Department of Psychiatry, Psychotherapy and Psychosomatic Medicine, Vivantes Hospital Am Urban und Vivantes Hospital im Friedrichshain, Charité University Medicine Berlin, Berlin, Germany; 4 Department for Psychiatry and Psychotherapy, Charité University Medicine Berlin, Berlin, Germany; 5 Centre for Psychiatry Suedwuerttemberg, Zwiefalten, Germany; 6 Gemeinnützige GmbH für Psychiatrie Reutlingen (PP.rt), Academic Hospital of Tuebingen University, Reutlingen, Germany; 7 Department of Psychiatry and Psychotherapy, Brandenburg Medical School Theodor Fontane, Immanuel Hospital Rüdersdorf, Rüdersdorf, Germany; 8 kbo-Isar-Amper-Klinikum, Region München, Munich, Germany; 9 Competence Center for Clinical Trials Bremen, University of Bremen, Bremen, Germany; 10 Centre for Psychiatry Suedwuerttemberg, Ravensburg, Germany; 11 Department of Psychiatry and Psychotherapy I, Ulm University, Ravensburg, Germany; 12 Department of Psychiatry, Psychotherapy and Psychosomatic Medicine, Vivantes Hospital Neukölln, Berlin, Germany; 13 Center for Psychiatry Reichenau, Reichenau, Germany; 14 Department of Psychiatry and Psychotherapy, University Hospital Tuebingen, Tübingen, Germany; 15 Department of Psychiatry and Psychotherapy II, BKH Günzburg, Ulm University, Günzburg Germany; 16 Research Institute of the Local Health Care Funds (WIdO), Berlin, Germany; 17 Bezirksklinikum Mainkofen, Deggendorf, Germany

**Keywords:** crisis resolution team, evaluation, home treatment, psychiatric services, severe mental illness

## Abstract

**Background:**

Inpatient equivalent home treatment (IEHT), implemented in Germany since 2018, is a specific form of home treatment. Between 2021 and 2022, IEHT was compared to inpatient psychiatric treatment in a 12-months follow-up quasi-experimental study with two propensity score matched cohorts in 10 psychiatric centers in Germany. This article reports results on the treatment during the acute episode and focuses on involvement in decision-making, patient satisfaction, and drop-out rates.

**Methods:**

A total of 200 service users receiving IEHT were compared with 200 matched statistical “twins” in standard inpatient treatment. Premature termination of treatment as well as reasons for this was assessed using routine data and a questionnaire. In addition, we measured patient satisfaction with care with a specific scale. For the evaluation of patient involvement in treatment decisions, we used the 9-item Shared Decision Making Questionnaire (SDM-Q-9).

**Results:**

Patients were comparable in both groups with regard to sociodemographic and clinical characteristics. Mean length-of-stay was 37 days for IEHT and 28 days for inpatient treatment. In both groups, a similar proportion of participants stopped treatment prematurely. At the end of the acute episode, patient involvement in decision-making (SDM-Q-9) as well as treatment satisfaction scores were significantly higher for IEHT patients compared to inpatients.

**Conclusions:**

Compared to inpatient care, IEHT treatment for acute psychiatric episodes was associated with higher treatment satisfaction and more involvement in clinical decisions.

## Introduction

Psychiatric home treatment (HT) or Crisis Resolution Team (CRT) care is widely accepted as an evidence-based alternative to inpatient care for a substantial proportion of people otherwise admitted to psychiatric inpatient care [1–3]. HT has been shown to reduce hospital admission rates [4, 5], without worsening clinical or social parameters [6]. Some studies showed that HT is better received than inpatient care, with higher rates of satisfaction with care in patients as well as caregivers [2].

Although the true potential of HT to reduce bed occupancy rates and to handle “difficult-to-treat-patients” [7, 8] has always been the subject of critical discussion, the compelling initial evidence for HT lead to further studies which generally replicated the clinical and readmission results of the British and US studies [9, 10].

Contrarily to many English-speaking countries, HT has until recently not been a national movement or a patient right in Germany. After a decade of a very limited offer of selected HT opportunities in a small number of regions, generally within time-limited pilot projects of flexible integrated care with HT components [11, 12], there has been a strong dynamic in establishing more HT/CRT services in the last years. Psychiatric HT is now fully reimbursed by the German social health insurance system since 2018. As inpatient equivalent home treatment (IEHT) or stationsäquivalente Behandlung (StäB), it can be implemented by single stand-alone teams offering HT or as assertive outreach carried out by a team which also has inpatient responsibilities [13]. With major characteristics of the original model of HT being shared such as regular home visits, low caseload, comprehensive psychiatric and medical assessment at home, common responsibility for medical and social care, intensive support, family/carer support, and crisis plans [14], German IEHT differs from British, US, Dutch or other HT models by its high degree of formalization in certain areas with the risk of financial loss in case of insufficient fulfillment of standards. In particular, contrary to the original British and some other models [1, 9, 14], daily home visits as well as contacts to a team psychiatrist twice a week and multiprofessional team sessions with a psychiatrist, a nurse, and at least one staff of another profession once a week are mandatory, as well as the possibility to offer inpatient admission at any time in the very hospital providing IEHT [15]. However, formal collaboration with the community mental health system is only optional. Evidence suggests that the requirement to offer daily visits without taking into account or allowing adaptions to regional peculiarities makes it more challenging to implement IEHT in rural catchment areas [16, 17]. As there is a high variability of HT models worldwide with different reimbursement as well as service delivery models, and since realizing its effectiveness requires specific key components [18], it is obvious that IEHT as specific German version of HT requires its specific evaluation building on the experience of other implementation models.

Against this background of a lack of solid evidence for IEHT in Germany, we present short-term results of a publicly funded quasi-experimental study comparing the German version of IEHT with inpatient care [19]. We focused on drop-out rates, patient satisfaction, and involvement in decision-making, because we expected IEHT to improve the therapeutic relationship resulting in measurable effects compared to standard hospital care.

Drop-out of treatment may be regarded as an extreme form of a broken therapeutic relationship or lack of adherence. Patient as well as caregiver satisfaction have been included as outcome variables in a variety of HT studies [20]. Randomized [6] as well as matched control studies [21] showed that most patients who have a choice and selected HT are more satisfied. Initial studies in German-speaking countries confirmed this trend concerning the new form of IEHT in German standard care [22]. This is in line with the finding that, in general, people with mental health crises and psychiatric diagnoses tend to be more satisfied with treatment in the community than in a psychiatric hospital [23].

Besides experiencing more autonomy, the reasons for higher satisfaction rates in home-based care, may include higher involvement in treatment decisions. Instruments measuring shared decision-making [24] can therefore assess this important aspect which might also have an impact on adherence to treatment procedures and jointly decided agreements.

Against this rationale, we combined these three outcome measures of therapeutic relationship to assess the effects of IEHT as a special form of HT/CRT care compared to inpatient treatment of acute psychiatric episodes. We hypothesized that IEHT is associated with higher satisfaction, more involvement in treatment decisions, and less premature termination of treatment compared to inpatient care.

## Methods

### Study design

We report results based on a multisite, pragmatic, quasi-experimental study which examined the implementation, treatment processes, clinical efficacy, and costs of as well as subjective experiences in IEHT compared to inpatient treatment from the perspective of service users, relatives or informal caregivers, staff and other stakeholders in mental health care. The details of the protocol of the “AKtiV” study (“Aufsuchende Krisenbehandlung mit teambasierter und integrierter Versorgung”) are outlined in a Methods paper [19]. In short, the study was run in 10 psychiatric centers in Southern and Eastern Germany where IEHT has been implemented recently. Sites were from both urban (center of Berlin with three, Berlin1, Berlin2, and Berlin3, and center of Munich with one site) and rural areas (Brandenburg located north-east of Berlin with one center in Rüdersdorf as well as five centers in rural or small-town regions in South-Western Germany, Baden-Württemberg: Reichenau, Reutlingen, Tübingen, Weissenau, and Zwiefalten). All centers had defined catchment areas with full mental health service responsibility. IEHT implementation had been started in the study sites between July 2018 and August 2020. The trial was conducted between 2021 and 2022.

Each center steered the recruitment process separately with support from the coordinating site (Berlin). Patients participating in routine IEHT in each center were consecutively asked for study participation. All patients included in IEHT during the recruitment period fulfilling the trial inclusion criteria were asked for participation. Each participant received a propensity score (PS) predicting the probability of receiving IEHT in the specific study center. The PS was based on the total number of psychiatric inpatient treatments or IEHT in the study center in the last 2 years, the main psychiatric diagnosis, age, and gender.

For each included participant in the IEHT arm, a patient match from the pool of recently admitted regular inpatients fulfilling IEHT inclusion criteria was built, based on the most similar propensity score. Thus, we created comparable twins with similar clinical and sociodemographic characteristics. For more details and sample size calculation see the study protocol [19].

#### Intervention and control treatment

IEHT treatment is team-based home care provided by a team of psychiatrists, psychologists, psychiatric nurses, social workers, and other professions at the discretion of the study centers. The IEHT treatment processes have been defined in detail by the umbrella organization of all German social health insurance companies and the German Hospital Society [25]. Reimbursement of IEHT by the German statutory health insurance is subject to certain requirements: There must be an indication for inpatient treatment (a psychiatric crisis) to be allowed to receive IEHT, a psychiatrist must assess IEHT suitability (adequacy of the home, enough privacy for encounters at home), a written treatment plan must be made, the team must be available continuously 7 days a week for the whole day (at night, generally the psychiatrist at the emergency department of the hospital and the psychiatrist on-call must be available, not a genuine team member), at least one team member must realize one daily face-to-face contact every day, the team has to meet once a week to discuss each patient in detail, and individual contacts must be documented (content and exact time).

The IEHT team is responsible for the whole psychiatric care as well as somatic issues, including diagnostics, medications, psychotherapy, and social issues. During the first days, an individual needs assessment is done. The treatment plan builds on that. This plan includes treatment goals, various measures such as medication, psychotherapy, training, and other daily or therapeutic activities, as well as therapeutic interactions with relatives, informal caregivers, legal guardians, and other persons from the participant’s social network. Therapeutic interventions are adapted to the users’ needs on a daily basis. Daily personal contacts can either take place at home, at the hospital, or at any place the service user felt comfortable with. However, at least six encounters per week with service users are realized outside of the hospital. In the weekly consultation by the psychiatrist in charge, the treatment progress is reflected upon, and further interventions are planned. Treatment is implemented according to the available resources and standards of the study sites. For each patient, treatment-related questions are discussed extensively in regular, inter-professional IEHT team meetings at least once a week. They involve medical staff, nurses at least one psychologist, one social worker, or one member of another profession. Treatment is finished only after extensive discharge planning.

Inpatient treatment was done according to the standards of psychiatric hospitals in Germany.

#### Assessment and outcome measures

At baseline, demographic and the following psychometric variables were assessed. Symptom severity was measured by the German version of the Health of the Nations Outcome Scale (HoNOS-D) [26], psychosocial functioning measured by the German version of the Personal and Social Performance Scale (PSP) [27], quality of life measured by the EQ5D index [28], and Recovery Orientation was measured by the German version of the Recovery Assessment Scale (RAS-G) [29].

For the analyses of the acute treatment episode during which participants were included in the study (index episode), we report three outcome measures: premature treatment discontinuation, patient satisfaction, and experienced shared decision-making.

Premature discontinuation during the index episode as well as reasons for this was assessed independently by one scientific researcher at each site. We used information sent by the treatment team (IEHT or inpatient) as well as routine data collected by the hospitals. Reasons for treatment discontinuation could be: (1) *external reasons* unrelated to treatment such as patients moving to a place outside the catchment area, loss of the home, medical problems requiring hospital admission or referral, or death unrelated to treatment; (2) *patient dropping out on their own against doctor’s advice*; and (3) *treatment failure* such as severe noncompliance resulting in discharge or insufficient IEHT treatment requiring direct inpatient admission.

Patient satisfaction with care was measured using a special 18-item Likert scale developed in one of the participating centers [22]. The scale was based on a quality management instrument (TÜBB 2000) used to assess satisfaction of care with psychiatric treatment [30]. Each of the 18 items could be answered with the following options: (1) strongly agree – 100 points, (2) agree – 75 points, (3) undecided – 50 points, (4) disagree – 25 points, and (5) strongly disagree – 0 points. A higher average total score equals higher satisfaction.

The evaluation of patient involvement in treatment decisions was performed using the 9-item Shared Decision Making Questionnaire (SDM-Q-9), a validated scale to evaluate shared decision-making at the end of an index episode [31]. The SDM-Q-9 is a patient-reported measure that focuses on the decisional process by rating physicians’ and patients’ behavior in medical encounters. It was developed as a revision of the original Shared Decision-Making questionnaire [32]. The 9-items-scale was adjusted from 4-point to 6-point ratings with extremes (“0 = completely disagree” to “6 = completely agree”) to counter high ceiling effects [31]. The possible overall sum score thus ranges between 0 and 45. The sum scores have to be multiplied with 20/9 to reach an end score between 1 and 100 with higher scores indicating more information for the service user and more involvement in treatment decisions such as more common weighing of joint selection between treatment options. The SDM-Q-9 showed good internal consistency (*α* = 0.94) as well as high face and structural validity in its first psychometric testing in a large (*N* = 2,351) primary care sample [31].

#### Data analysis

Analyses between baseline data as well as between all three outcome parameters were performed on an intention-to-treat basis, including all patients initially participating. For each analysis of outcome parameters, only those patients with observed values for all variables under consideration were included (complete case analysis). The number of patients included is reported.

Statistical analyses were conducted with SAS Version 9.4 (TS1M3) and SYSTAT 13.2 as well as programs based on this statistical package. Descriptive analyses were conducted for all quantitative data. For all categorical variables, numerical and percentage data were calculated separately for the IEHT and for the inpatient group. For metric variables, mean, standard deviation, median, minimum, and maximum were calculated additionally.

For subgroup analyses, the IEHT intervention and the inpatient control group were subdivided as follows: IEHT-A: direct admission to IEHT without previous inpatient treatment; IEHT-B: IEHT right after inpatient treatment (bridging to outpatient care or shortening of an inpatient stay); control patients for both groups were put together in the inpatient group.

To evaluate the equivalence of intervention and control group in key demographic variables balance, we performed Mann–Whitney’s test for age and number of psychiatric inpatient treatment episodes during the last two years, Chi-squared test for gender, and Fisher’s exact test for diagnosis. Outcomes for the index episode were tested in an explorative manner with *α* = 5%. Differences in premature treatment discontinuation in the index episode were tested using Chi-squared test or, if one cell count was less than 5, by Fisher’s exact test. When comparing two groups, Mann–Whitney’s test was used for the outcome parameter shared decision-making and Welch’s test for treatment satisfaction. For more than two groups, Kruskal–Wallis’ test was used.

## Results

### Participants

A total of 1367 patients were screened for inclusion with a total of 400 persons being finally included (200 receiving IEHT and 200 inpatient treatment as usual). The participants were distributed among the centers as follows: *n* = 58 Berlin1, *n* = 36 Berlin2, *n* = 26 Berlin3, *n* = 40 München, *n* = 38 Rüdersdorf, *n* = 50 Reichenau, *n* = 50 Reutlingen, *n* = 8 Tübingen, *n* = 50 Weissenau, and *n* = 44 Zwiefalten. *N* = 144 (72%) of all IEHT patients were direct IEHT admissions (IEHT-A), and *n* = 56 (28%) received IEHT right after inpatient treatment (IEHT-B).

Two-thirds of the whole sample (*n* = 264; 66%) were female, the mean age was 45 years (Table 1). Most patients had a diagnosis of recurrent depression, a manic episode within a bipolar disorder or a psychotic episode. The mean number of psychiatric inpatient treatment episodes or IEHT in the study center during the last two years was 1.6 in the IEHT group and 1.2 in the inpatient group. There was no significant difference between the highest professional training achievement with 39.5% having gone through a vocational training, 19.0% holding a university degree, and 22.8% with no formal professional training. More patients in the inpatient group current had a job, compared to the IEHT group. Besides this, there were no differences between the both groups for baseline parameters.

**Table 1.** Patient characteristics IEHT compared to inpatient treatment, *n* = 400

Item		IEHT (mean/frequency)	Inpatient treatment (mean/frequency)	Test statistic	*p*
Age	Years	45.3	45.6	Mann–Whitney	n.s.
Sex: female	*n* (%)	136 (68.0)	128 (64.0)	Chi-square	n.s.
Number of previous inpatient stays		6.7	5.3	Mann–Whitney	n.s.
Marital status: married or partnership	%	45.5	34.5	Chi-square	n.s.
Without professional education	%	21.5	24.0	Fisher test	n.s.
Currently with a job	%	44.0	54.0	Chi-square	n.s.
Age at first inpatient treatment		34.8	34.9	Mann–Whitney	n.s.
Symptom severity (HoNOs)	Mean (SD)	14.7 (5.4)	15.3 (5.5)	Mann–Whitney	n.s.
Psychosocial functioning (PSP)	Mean (SD)	56.0 (12.3)	56.1 (13.0)	Mann–Whitney	n.s.
Quality of life (EQ5D)	Mean (SD)	0.62 (0.30)	0.64 (0.28)	Mann–Whitney	n.s.
Diagnoses					
Organic disorder	*n* (%)	1 (0.5)	0		
Substance disorder	*n* (%)	13 (6.5)	13 (6.5)		
Substance disorder	*n* (%)	43 (21.5)	43 (21.5)		
Affective disorder	*n* (%)	94 (47.0)	95 (47.5)		
Neurotic disorder	*n* (%)	31 (15.5)	31 (15.5)		
Other behavioral syndromes	*n* (%)	2 (1.0)	2 (1.0)		
Personality disorder	*n* (%)	16 (8.0)	16 (8.0)		

Abbreviations: HoNOS, Health of the Nations Outcome Scale; IEHT, inpatient equivalent home treatment; PSP, Personal and Social Performance Scale.

Symptom severity as measured by the Health of the Nations Outcome Scale (HoNOS) was moderate and comparable between both groups. The same holds true for psychosocial functioning as measured by the Personal and Social Performance Scale (PSP) and for quality of life as measured by EQ5D index. There were no significant differences between clinical parameter between both groups either.

On average, patients spent 37.2 days in IEHT treatment while inpatient treatment comprised on average of 28.2 days. There was considerable variation between the center means with a minimum IEHT duration of 26.5 days in one center and a maximum of 91.5 days in another center.

With regard to diagnoses, across all centers, people with primarily organic psychiatric disorders (*n* = 1; 13.0 days) or personality disorders (*n* = 16; 27.3 days) spent considerably less time in IEHT than people with psychotic, affective, neurotic, or eating disorders (Table 2).

**Table 2.** Duration of index episode according to primary psychiatric diagnosis

Duration of treatment (days)		*N*	Mean (SD)	Min	Max
					
Organic disorder	IEHT	1	13 (0)	13	13
	Inpatient	–	–	–	–
Substance disorder	IEHT	13	29.8 (24.2)	7	95
	Inpatient	13	16.5 (14.8)	5	63
Psychotic disorder	IEHT	43	37.8 (37.6)	6	232
	Inpatient	43	22.6 (19.0)	3	94
Affective disorder	IEHT	94	39.0 (19.2)	4	85
	Inpatient	94	32.7 (33.8)	2	265
Neurotic disorder	IEHT	31	38.2 (19.1)	10	96
	Inpatient	31	31.5 (38.7)	2	206
Other behavioral syndromes	IEHT	2	62.5 (12.0)	54	71
	Inpatient	2	61.5 (62.9)	17	106
Personality disorder	IEHT	16	27.3 (21.8)	7	95
	Inpatient	16	15.8 (10.9)	0	42
All diagnoses	IEHT	200	37.2 (24.9)	4	232
	Inpatient	199	28.2 (30.6)	0	265

Abbreviations: IEHT, inpatient equivalent home treatment.

### Premature treatment discontinuation in the index episode

In both groups, a similar proportion of patients discontinued treatment in the index episode (Table 3). Reasons for treatment discontinuation were: (1) external reasons (IEHT: *n* = 4; 28.6% vs. inpatient care: *n* = 5; 23.8%); (2) patients dropping out against doctor’s advice (IEHT: *n* = 4; 28.6% vs. inpatient care: *n* = 10; 47.6%); and (3) treatment failure (IEHT: *n* = 6; 42.9% vs. inpatient care: *n* = 6; 28.6%). Neither death nor other reasons for treatment termination occurred during the index episode.

**Table 3.** Premature treatment discontinuation during index episode (*N* = 400)

Treatment discontinuation		Yes	No	*p*
		*N* (%)	*N* (%)	
*Treatment group*				0.551^a^
IEHT		14 (7.0)	186 (93.0)	
Inpatient care		21 (10.5)	179 (89.5)	
*Sex (male)*		21 (15.4)	14 (5.3)	<0.001^b^
Total		35 (8.8)	365 (91.3)	

Abbreviations: IEHT, inpatient equivalent home treatment.

a IEHT versus inpatient care, Fisher test.

b Male versus female, chi-square test.

Within the IEHT group, participants with direct IEHT admission had a dropout rate of 7.6% (11 of 144), and participants with IEHT after inpatient care had a dropout rate of 5.4% (3 of 56).

In the combined analysis of all participants of both groups, treatment discontinuation rates did not vary significantly according to number of previous inpatient stays, employment status, and main psychiatric diagnosis. However, significantly more male than female patients stopped treatment prematurely.

### Satisfaction with treatment

Patient satisfaction during the treatment in the index episode was significantly higher for the IEHT compared to the inpatient group (Table 4).

**Table 4.** Patient satisfaction in IEHT compared to inpatient treatment^a^

Patient satisfaction		*N*	Mean (SD)	Min	Max	*p*
	IEHT	187	81.1 (16.8)	19.4	100	
	Direct IEHT admission	134	82.0 (16.4)	22.2	100	
	IEHT after inpatient care	53	78.9 (17.8)	19.4	100	
	Inpatient	173	73.2 (17.9)	14.7	100	<0.01^b^
Total		360	77.3 (17.8)	14.7	100	

Abbreviations: IEHT, inpatient equivalent treatment; SD, standard deviation.

a Measured with an 8-items instrument, score range of 0–100.

b IEHT versus inpatient care, Welch’s test.

The main analysis with the full-case sample using Welch’s test yielded statistically significant higher satisfaction scores for patients in the IEHT group (*p* < 0.001; estimate 7.95, df = 350.6). Compared to the inpatient group, both the subgroup of patients with direct IEHT admission (*p* < 0.001) as well as those receiving IEHT right after inpatient treatment (*p* < 0.05) had significantly higher scores concerning satisfaction with treatment. In the combined analysis of all participants of both groups, patient satisfaction did not vary significantly according to sex, number of previous inpatient stays, employment status and main psychiatric diagnosis.

### Shared decision-making

Patients in IEHT felt significantly more involved in their treatment and gave significantly higher ratings in the SDM-Q-9 measure than inpatients (Table 5).

**Table 5.** Patient involvement in care (shared decision-making), index episode, SDM-Q-9

SDM-Q-9		*N*	Mean (SD)	Min	Max	*p*
	IEHT	150	69.6 (24.2)	4.4	100	
	Direct IEHT admission	104	70.7 (25.3)	4.4	100	
	IEHT after inpatient care	46	67.3 (21.7)	11.1	100	
	Inpatient	144	59.9 (26.9)	0	100	<0.01^a^
Total		294	64.9 (26.0)	0	100	

Abbreviations: IEHT, inpatient equivalent home treatment; SD, standard deviation; SDM, shared decision-making.

a IEHT versus inpatient, Mann–Whitney’s test.

Compared to the inpatient SDM-Q-9 scores, only the subgroup of patients with direct IEHT admission had significantly higher scores concerning shared decision-making. In the combined analysis of all participants of both groups, SDM-Q-9 scores did not vary significantly according to sex, number of previous inpatient stays, employment status, and main psychiatric diagnosis.

## Discussion

### Main findings

Our results show that, while treatment discontinuation rates were similar for both groups, patients were significantly more satisfied with IEHT than with inpatient treatment. People in the IEHT group also felt more involved in treatment decisions yielding higher shared decision-making score ratings.

### Strengths and limitations

This is the first study assessing the German model of HT/CRT called IEHT. It is the first study evaluating shared decision-making during IEHT compared to inpatient treatment. Included patients were from different regions in Germany. As IEHT is strictly standardized with regard to its main processes, the model was similar in all sites.

Despite all efforts to create comparable treatment groups, selection effects cannot be ruled out to account for some results. The study was not a randomized controlled study but used the second-best alternative, propensity score matching. Besides our four propensity score variables, other clinical variables with a possible influence on the outcome could not be applied. Thus, patient in the IEHT group may have had a higher amount of autonomy (which was required to be treated at home) and more preferences to actively take part in their own treatment. In addition, caregiver involvement is more easily achieved during home treatment compared to inpatient treatment in psychiatry [33]. It is probable that during home treatment barriers for SDM were lower on both sides.

We did not included patients which were involuntarily admitted [34], thus our results are only valid for those with a self-declared need for psychiatric help.

In some subgroup analyses such as those comparing IEHT after inpatient care (as opposed to direct IEHT admission versus inpatient care) the numbers were moderate thus maybe increasing the probability of a type 2 error although we accounted for the small sample size by testing premature treatment discontinuation with Fisher’s exact test if a cell count was less than five.

### Interpretation of results

#### Treatment discontinuation

Rates of discontinuation in this trial were low both for IEHT as well as for inpatient care. The British as well as the recently published Dutch [10] and Swiss HT study [9] cannot be directly compared to our study with regard to treatment discontinuation as they measured discontinuation rates during the 12-months follow-up without focusing on the index episode. A large evaluation of a German health insurance company yielded an inpatient discontinuation rate of 8.9% for psychiatric wards (Techniker Krankenkasse, 2011, personal communication) comparable to our results. Taking into account a general high preference of many people experiencing a psychiatric crisis to be treated in the community or at home [20, 23], we would have expected lower drop-out rates in the IEHT group compared to hospital treatment. As treatment discontinuation is a complex issue with interactions between patient as well as treatment factors, drop-out rates may not indicate variances in preferences or satisfaction.

#### Satisfaction and acceptance

Our study could replicate the findings of international, particularly British, studies of high acceptance rates of HT/CRT [6, 35, 36]. Similar findings could be seen in a qualitative evaluation with a subsample of a large randomized controlled study in Switzerland [37]. This qualitative study could identify special individualized treatment, more exclusive time for each patient, practical support for patients’ daily routines, and more self-efficacy as important factors for higher satisfaction rates compared to hospital treatment [37].

Our results with regard to higher satisfaction, however, are not in line with the two randomized studies conducted recently in Switzerland and the Netherlands which did not find any differences in satisfaction scores after intensive home treatment compared to inpatient care [9, 10]. However, the scale used in the Dutch study was not validated in people with psychiatric disorders [38]. The same is true for the Perception-of-Care scale PoC-18 which was used in the Swiss study without a scientific reference. These scores may have lacked some sensitivity to change in people with severe psychiatric disorders. An alternative explanation for our more positive results may be our study not having been a randomized one with participants in IEHT potentially having had a more positive attitude toward the team or having been more grateful not to have to be admitted to hospital resulting in more satisfaction.

#### Shared decision-making

Higher perceived involvement in decision-making might in part be a result of practical support and more self-efficacy in the IEHT arm. There is, however, no evidence that home treatment compared to hospital care differentially increases self-efficacy, at least using the Mental Health Confidence Scale [39]. The Dutch HT study which is the only one using this outcome measure found no general effects on self-efficacy concluding that it might not be reasonable or feasible to empower self-efficacy during an acute psychiatric crisis but rather later at follow-up [40]. We used neither a recovery scale nor a self-efficacy scale, therefore more research is needed whether satisfaction and perceived involvement in decision making in acute mental health crises are separate constructs or have aspects of interdependency.

One could argue that home treatment requires different forms of patient-provider interaction than inpatient treatment. Home treatment requires more negotiation, as team members are only present at the patient’s home for 1–2 hours a day and patients have to adhere to (shared) decisions during the rest of the day without further support or advice. In addition, HT members enter the patient’s home which itself requires other modes of communication and other roles than at hospital. Therapists are thus less able or less inclined to impose their narrative of the patients’ problems on them and to act as an expert or authority within a hierarchy of “knowing” better than the service user [41]. It is likely that HT staff members are more prone to cooperative decision-making styles than inpatient staff. On the other side, patients’ competence to actively engage in decision making and finally adhering to what has been decided jointly with the IEHT team might have been a selection criterion for IEHT (with less adherent patients potentially being more likely to be admitted to inpatient units).

Another factor promoting SDM in the IEHT group may have been the presence of caregivers during home treatment. During HT, mental health professionals may involve relatives and other caregivers not only by informing them and making efforts to get their support for decisions which the clinicians consider to be adequate [42], but by finding solutions patients as well as relatives can live with. Outside the hospital, caregivers may find it easier to articulate their preferences when they are in their own social context or are invited by the patients themselves rather than by the clinicians. In addition, taking into account that staff members need support, training, and education to apply SDM involving caregivers [43], HT may provide more opportunities of on-the-job training to involve relatives in patients’ homes.

### Implications and conclusion

Since IEHT as a specific form of HT is highly acceptable, it should be a central part of a psychiatric care system. At the same time, more research should be done with regard to the target population and the true potential to substitute inpatient admissions. It should also be evaluated if shared decision-making is facilitated by the model or if some SDM preferences are required for successful IEHT/HT.

## Data Availability

The data that support the findings of this study are available from the corresponding author upon reasonable request.

## Trial registration

Trial registrations: (1) German Clinical Trials Register (DRKS), DRKS000224769. Registered December 3, 2020, https://www.drks.de/drks_web/setLocale_EN.do; (2) Clinicaltrials.gov, Identifier: NCT0474550. Registered February 9, 2021, https://clinicaltrials.gov/ct2/show/NCT04745507.
